# Recent Advances and Applications of Passive Harmonic RFID Systems: A Review

**DOI:** 10.3390/mi12040420

**Published:** 2021-04-12

**Authors:** Saikat Mondal, Deepak Kumar, Premjeet Chahal

**Affiliations:** Department of Electrical and Computer Engineering, Michigan State University, East Lansing, MI 48823, USA; mondalsa@msu.edu (S.M.); kumarde2@msu.edu (D.K.)

**Keywords:** RFID, harmonic, NLTL, clutter

## Abstract

Harmonic Radio Frequency Identification (RFID) systems have attracted significant interest over the last decade as it provides many benefits over the conventional RFID systems. Harmonic RFID is desired over conventional RFID systems due to reduced self-jamming, location accuracy from dual frequency, and higher phase noise immunity. In a harmonic RFID system, the tag receives instructions from the reader at an RF carrier frequency and replies back at the harmonic of the RF frequency. A nonlinear element consuming very low power at the tag is required to generate the harmonic carrier for the battery-less system. In this review article, a detailed contrast between conventional and harmonic RFID systems is presented. This is followed by different circuit design techniques to generate harmonics and integration techniques to form a fully operable passive harmonic RFID tag. Also, a wide range of applications, especially sensor integration with harmonic RFID’s, along with the future trends are presented.

## 1. Introduction

Radio Frequency Identification (RFID) technology has emerged as a successful technology for tagging, tracking, sensing, and locating objects [1,2,3] in different sectors such as healthcare, retail, logistics, and agriculture [4,5,6]. RFIDs can work at different frequencies, starting from a low frequency (LF) band to microwave ISM (Industrial, Scientific, and Medical) bands with or without on-board power source such as batteries. Active RFIDs are battery powered [7], whereas passive type RFIDs harvest energy from the incoming Electromagnetic (EM) field. Active RFIDs can have a very long read range of 100 m and longer, in some cases, more than 1 km [8,9], whereas the read range of a passive RFID is limited by their energy harvesting capability [10]. A passive LF RFID is used for short range communication (∼5 cm), whereas the ultra high frequency (UHF) passive RFID has a longer read range (∼6–8 m). The exclusion of batteries in passive RFIDs allows them to have a small form factor and leads to lower cost, and makes them easier to adopt for many applications. Advances in RFID have been possible due to state-of-the-art CMOS fabrication technology, development of printing techniques for low cost and bulk manufacturing, and development of required software for scalable business operation [11]. The RFID market has become a multi-billion dollar industry and continues to grow.

Beyond object tagging, demand for RFIDs is increasing in many other applications such as sensing and localization. Food packages tagged with RFID-based sensors can help in dramatically reducing food wastage by seamless monitoring of food quality across the food supply chain [12,13]. Another important growing application of RFID is in the localization of products. Localization of specific RFID tagged objects in retail stores, sorting facilities, warehouses, and resource management facilities can automate the handling process and minimize the possibility of misplaced items [14,15,16]. Due to a shorter read range of passive RFIDs, an unmanned vehicle with an RFID reader has been proposed to localize desired tagged objects at large facilities for complete automation [14,17,18]. Usually, the received signal strength indicator (RSSI) and phase of return RF signal are measured by a conventional RFID reader to estimate a tag’s location. This method does not provide good accuracy, especially when many tags are co-located. Furthermore, reflection from nearby scatterers, such as metal racks and shelves, can induce multipath fading and hence degrade the localization accuracy. To overcome this challenge, different measurement methods such as spatial-temporal phase profiling (STPP) for relative object localization [19], Bayesian filter, and a variable power RFID model (BFVP) [20] have been proposed for improved localization accuracy. Those methods were demonstrated in complex real-world applications such as locating objects in a mock apparel store, misplaced books in a library, or determining the baggage order at an airport.

Apart from the multipath effect, background clutter is a major challenge for RFID technology as it reduces the desired read range. Clutter is background reflection from nearby objects and it can overshadow the desired signal coming from the RFIDs and thus degrade the signal-to-noise ratio (SNR) at the reader [21]. In addition, strong clutter can significantly reduce the localization accuracy [22]. Most of the proposed solution to reduce the clutter and multipath effects are dependent on heavy computation at the reader and in some cases requires a detailed understanding of the operating environment. Recently, a new type of RFID, harmonic RFID, was proposed to mitigate the clutter and localization challenges [23,24]. In contrast to the conventional RFID system, the harmonic RFID system uses two different frequencies (fundamental and its harmonic) for down- and up-link communication between the RFID reader and tag. This review article presents the fundamental advantage, progress, applications, and future harmonic RFID trend.

This review article is arranged as follows: In Section 2, the drawback of conventional RFID and the advantage of harmonic RFID is analyzed and presented. In Section 3, the advantages and disadvantages of different nonlinear devices in generating harmonics are compared. In Section 4, an enhanced harmonic generation architecture is presented, using the nonlinear devices presented in the previous section. In Section 5, different system level design of completely developed harmonic RFID tags in literature are discussed. In Section 6, applications of harmonic RFID in different sectors are presented. Finally, in Section 7, the current and future trends with harmonic RFID challenges are described.

## 2. Drawback of Conventional RFID

Although conventional RFID has many advantages, there are also certain drawbacks that arise due to the fundamental operating principle of RFID. The primary two drawbacks of conventional RFID are: (a) clutter, and (b) localization. The drawbacks are discussed first from a system operation point of view and the advantage of harmonic RFID in mitigating those drawbacks is elaborated later.

### 2.1. Clutter

**Figure 1:**
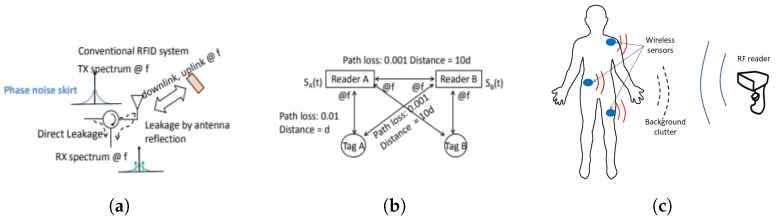
Different sources of clutter (**a**) self-jamming [25], (**b**) multi-reader jamming [25], [Reproduced with permission from Y. Ma, A passive broadband harmonic Radio Frequency Identification (RFID) platform; published by IEEE, 2016.] (**c**) Reflection from human body in a body area network (BAN) sensor [27]. [Reproduced with permission from S. Mondal, Scope and application of harmonic RFID for implanted body area network; published by IEEE, 2020].

Clutter is described as an unwanted signal, which can obscure the desired signal and hence diminish the system performance. The clutter source in conventional RFID can arise from several places: (1) self-jamming due to mismatch at the antenna port, (2) multi-reader jamming when multiple RFID readers are transmitting simultaneously and one can jam the other, (3) reflection from nearby objects [25,26,27]. The primary clutter objects are usually different in different applications. For example, the primary clutter object for a body area network (BAN) based sensor or sensor monitoring body vitals is the biological body [28]. In underground object tagging, the ground is a source of strong clutter [29,30]. Similarly, in industrial environments, metal objects are a source of clutter [24]. The different clutter sources are shown pictorially for multiple scenarios in Figure 1.

**Figure 2:**
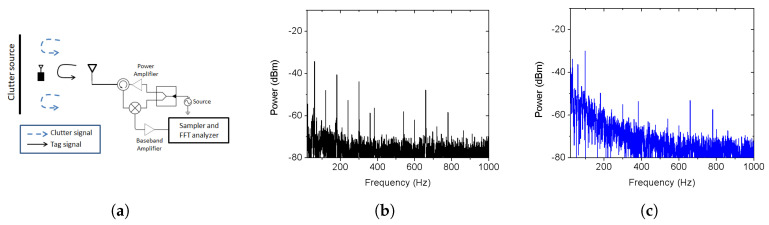
(**a**) The reader circuit to measure the clutter, (**b**) clutter phase noise in presence of an absorber, and (**c**) clutter phase noise in the presence of a clutter source [27]. [Reproduced with permission from S. Mondal, Scope and application of harmonic RFID for implanted body area network; published by IEEE, 2020].

In [24], a mathematical formulation is provided to show how the clutter phase noise can overshadow the low power tag return modulation signal. Furthermore, experimentally, it was demonstrated that a conventional RFID read rate reduces in the presence of a clutter source. The read rate was directly correlated to the signal-to-clutter ratio (SCR), and hence, the read rate can be analyzed to monitor the clutter effect. Clutter that originated due to reflection from the metallic scatterer was measured in [27] using the conventional RFID front end. In the reader front end, the baseband amplifier should be of very low noise to measure the system phase noise [31]. If the tag returned signal is of very low power and low frequency, it can get buried under the clutter phase noise. The reader circuit and the measured phase noise response in the absence and presence of the clutter source are shown in Figure 2.

### 2.2. Localization

RFID has become widely accepted in enterprise supply chain management systems as it improves the efficiency of inventory tracking. In the supply chain, RFID tagged object localization using RF signal is a desired feature. The RF localization is primarily done using RSSI, or phase information, or a combination of both [32,33]. However, precise localization in a conventional RFID system is a challenge due to (a) multipath effect, (b) undesired interference, and (c) presence of multiple RFID tags [34]. In RSSI-based localization, the signal attenuation level is monitored and the distance is estimated from Friis transmission equation. The multipath effect is stronger in this method due to the received signal power level measurement. In phase-based measurement, the phase of the arrival of the backscattered signal is measured. However, accurate phase-based localization is possible when the bandwidth is wider, which is difficult for a conventional RFID system with only 30 MHz bandwidth. Interference among multiple readers is possible and can contribute to distance uncertainty. Furthermore, when the RFID tags are densely populated, it becomes difficult to remove localization ambiguity among the tags. The inherent problem lies in the frequency of operation and the localization ambiguity reduces with an increase in the operating frequency.

### 2.3. Harmonic RFID as a Solution

Clutter and localization issues can be substantially mitigated by employing a harmonic RFID solution. The harmonic RFID tag and reader operates at two different frequencies: (a) reader to tag downlink at the fundamental frequency, and (b) tag to reader uplink at the harmonic frequency. In a harmonic RFID, the tag generates harmonic of the reader transmitted RF signal and uses that as a carrier for modulation. Harmonic RFID has an advantage in a cluttered environment as the clutter appears at the fundamental frequency and the tag return signal is at the harmonic frequency. Hence, the clutter cannot obscure the desired signal, unlike the conventional RFID system.

**Figure 3:**
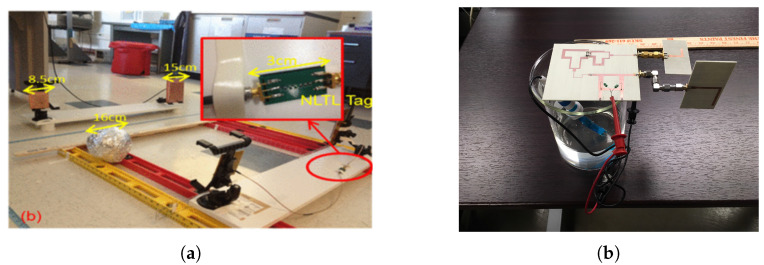
(**a**) Harmonic phase based localization in the presence of a scatterer [35] [Reproduced with permission from Y. Ma, Accurate indoor ranging by broadband harmonic generation in passive NLTL backscatter tags; published by IEEE, 2014]. (**b**) Harmonic phase-based sensing with better immunity to background clutter [36]. [Reproduced with permission from S. Mondal, A wireless passive pH sensor with clutter rejection scheme; published by IEEE, 2019].

Similarly, the localization is also improved when harmonic RFID is used. In [35], it was experimentally demonstrated that as the phase information is contained within the second harmonic, interferences and phase errors caused by direct reflections of the interrogating signal were greatly reduced. In addition, the phase information is better preserved in the harmonic operation and immune to background interference, as demonstrated in [36]. Simultaneously, as the readers transmit only the fundamental frequency, the reader-to-reader interference will be greatly reduced. Additionally, as the harmonic frequency reduces the operating wavelength, the localization accuracy is significantly enhanced [37]. Two different cases of harmonic phase based localization and sensing are shown in Figure 3.

## 3. Harmonic Generation

A nonlinear device, such as a diode or transistor, is needed to generate harmonics from an RF signal. A frequency multiplier is commonly used within a harmonic generator to produce the desired multiples of the input frequency. In the literature, different active or passive type frequency multipliers such as diode [23,29], nonlinear transmission line (NLTL) [24,38], transistor [39,40,41], or delayed lock loop (DLL) [42] were proposed to provide frequency multiplication from 2× to 8×. The relevant literature is summarized in Table 1 according to device type, multiplication factor, frequency range of operation, and power consumption. When an external power source such as a battery is required, the harmonic generator is the active type, otherwise it is the passive type. High DC power is required for the transistor-based harmonic generators at high frequency. On the other hand, diode-based harmonic generators consume almost zero or no DC power. Harmonic tags demonstrated in [23,29] use zero bias Schottky diodes and require no external DC power, and are the simplest design.

From Table 1, Schottky and varactor diode-based harmonic generators are ideal from a power consumption point of view due to very minimum or almost no power consumption. However, in addition to power consumption, other factors influence the harmonic tag design. Hence, different harmonic generation technologies are compared based on four key design factors: (a) conversion gain, (b) power consumption, (c) cut-off frequency, and (d) on–off keying.

### 3.1. Conversion Gain

**Figure 4:**
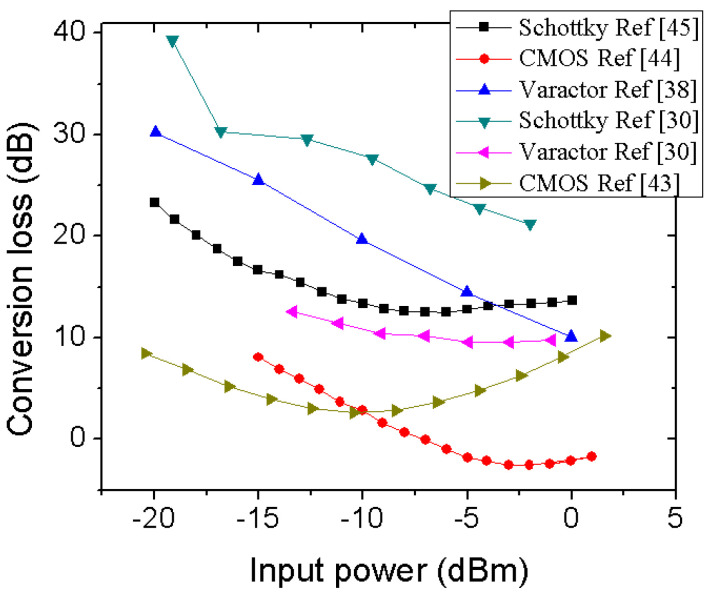
Conversion gain of harmonic generation using different technologies.

Conversion gain of a harmonic generator is usually defined as the power level difference between output harmonic power and input fundamental frequency power. A high value of conversion gain is desired to perform communication with a harmonic tag at a long range. Conversion gain of diode elements and a CMOS-based circuit is shown in Figure 4 at different input RF power levels. It can be noted that the CMOS-based technology shows the least conversion loss. However, the CMOS circuits use an intermittent amplifier stage to boost up the harmonic power level and suppress other undesired frequencies [43,44]. The Schottky diode, such as HSMS2850 in [45] and BAT1503 in [30], showed a decent conversion loss up to input power as low as −10 dBm. However, impedance matching and frequency of operation plays a key role in deciding the conversion loss in Schottky diode-based harmonic generators as pointed in [46], where it was demonstrated an improvement of 10 dB in conversion loss at −10 dBm input power when matching was performed. Two varactor diode-based harmonic generators were reported in [27,38], which shows good conversion loss at low input power. However, the operating frequency should be kept in mind while comparing conversion loss among different technologies. At high frequency, interconnects and package parasitics contribute to more loss resulting in poor conversion loss. Hence, monolithic fabrication is always desired while operating at a higher frequency. Due to the very small form factor, micro-fabrication-based technology shows very low parasitic or interconnect related loss at higher frequency. Hence, low conversion loss W or a higher band frequency doubler is not very uncommon using a monolithic process [47,48].

Harmonic generation is related to the device nonlinearity and influenced differently in CMOS or diode-based technologies. In general, the stronger the nonlinearity, the more efficient the harmonic generation, and, in turn, improved conversion loss will be observed. The nonlinearity factor is explored in different technologies as follows:

#### 3.1.1. Transistor-Based Harmonic Generator

An FET-based transistor needs to operate under the nearly pinched-off bias condition for a good nonlinearity and generate harmonics. A current reused frequency multiplier configuration was proposed in [44] using two FETs, where one FET was responsible for the harmonic generation and the other FET was used as an amplifier stage. As both the FETs are biased using the same DC current, the current reused frequency multiplier configuration consumes less power. Another popular architecture is Gilbert cell-based configuration, where nonlinearity from the FETs are utilized for harmonic generation [43]. Although power consumption rises, avoidance of inductors helps in realizing a smaller cell size for Gilbert cell-based multipliers. In general, the nonlinearity increases with an increase in drain current in a FET transistor. Hence, power consumption inherently increases in transistor-based harmonic generation for a good conversion loss.

#### 3.1.2. Diode-Based Harmonic Generator

**Figure 5:**
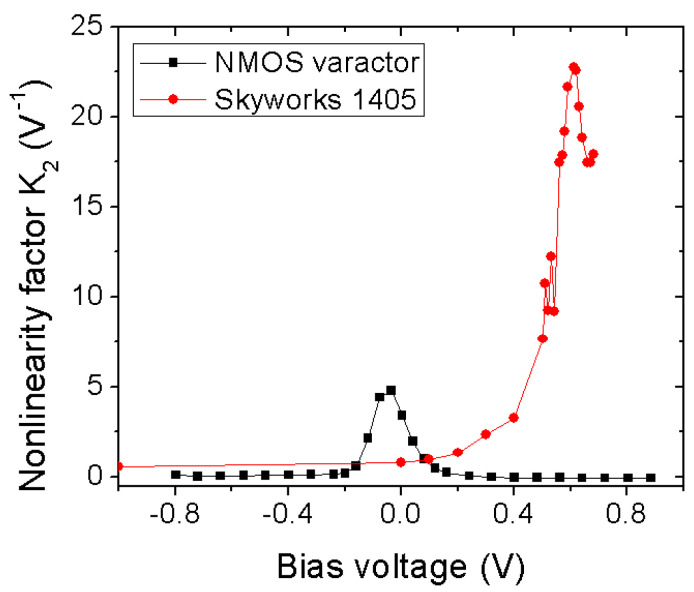
Second order nonlinearity factor ($K_{2}$) variation at different DC bias voltage.

Unlike a transistor, the nonlinear reactance of a diode is responsible for the harmonic generation. The junction capacitance of a diode is a function of bias voltage, as shown in Equation (1), where $C_{j0}$ is junction capacitance, $V_{bias}$ is the applied reverse bias voltage, $V_{j}$ is junction potential, and γ depends on the doping profile. The nonlinearity factor $K_{m}$ of a varactor diode can be extracted based on how the stored charge across the parallel plate behaves. As expressed in Equation (2), the diode can generate $m^{th}$ order harmonics [49] derived from stored charge, where $q(V_{bias})$ denotes the charge on diode capacitor at $V_{bias}$ and $q^{m}\left(V_{bias}\right)$ denotes the $m^{th}$ derivative of the charge at $V_{bias}$. Two different varactor diodes: (a) nMOS varactor from the IBM 8RF process [38], and (b) SMV1405 varactor from Skyworks [27] are compared for the second order nonlinearity in Figure 5. Figure 5 shows the second harmonic generation as a function of bias voltage. It can be noted that with stronger nonlinearity, the conversion loss improves at the respective operating frequency. Similar to a varactor diode, the nonlinearity factor of a Schottky diode is extracted from its nonlinear I-V characteristics. The nonlinearity of the Schottky diode is measured using the curvature factor, which is defined as the ratio of the $m^{th}$ derivative and first derivative of the current, similar to the charge ratio of a varactor diode as in (2) [48].

(1) $$C_{j}\left(V_{bias}\right)=\frac{C_{j0}}{{\left(1+V_{bias}/V_{j}\right)}^{\gamma }}$$

(2) $$K_{m}=\frac{q^{m}\left(V_{bias}\right)}{m!q^{\prime }\left(V_{bias}\right)}$$

### 3.2. Power Consumption

In passive harmonic RFID tags, power consumption is one of the major factors to select the harmonic generators from multiple options. Due to lack of battery, the passive harmonic RFID tags primarily depend on the RF harvested energy for desired operations. Hence, the harmonic generators’ power consumption should be kept as minimal as possible to conserve energy for other peripheral modules. To maintain strong nonlinearity, the transistor-based frequency multipliers need to operate at a higher operating current. This results in higher DC power consumption compared to diode-based multipliers. For example, a 1.6 to 3.2/4.8 GHz frequency multiplier in [40] requires 2.2 mW of external power at 1 V DC supply, and 250 μW from a 0.7 V supply for 1.2 GHz to 2.4 GHz multiplication in [41]. On the other hand, Schottky diodes usually have the maximum nonlinearity close to the threshold voltage, and due to very low threshold voltage, the Schottky diode can generate efficient harmonics at a bias very close to zero. However, a continuous DC path is required for the Schottky diode to maintain effective bias current for harmonic generation [29,45]. Simultaneously, it is important to consider the power consumption of a varactor diode at the bias condition for maximum nonlinearity. In literature, the reported varactor diode-based harmonic generators consume very little or almost no DC power.

### 3.3. Cut-Off Frequency

The cut-off frequency of harmonic generators is crucial as it dictates the maximum operational frequency range. The cut-off frequency primarily depends upon the design parameters, and the substrate used for active component fabrication. GaAs is desired over silicon substrate for high frequency operation due to increased electron mobility and saturation velocity. Apart from the substrate, parasitic components also reduce the device cut-off frequency. Hence, diode structure is preferred over transistor due to reduced parasitic capacitance. Naturally, it is common to find GaAs diode-based harmonic generators operating from hundreds of GHz to few THz [50,51]. In recent days, with significant process miniaturization down to 65 nm and beyond, it has become possible to realize frequency multipliers even beyond 100 GHz using silicon substrate [52,53].

### 3.4. Switching Control

Switching of the harmonic generator is required to perform digital modulation of the carrier. Harmonic generation in transistor- and varactor-based diodes can be easily controlled by voltage signal. On the other hand, the harmonic generation in the Schottky diode is controlled by the bias current. From an implementation point of view, voltage control is comparatively much easier than current control. Hence, the reported passive harmonic RFIDs in the literature are primarily realized using voltage-controlled varactor diodes.

Finally, the different harmonic generation techniques are summarized and compared in Table 2 from four different aspects. From the comparison, it can be observed that varactor diode-based nonlinear element is preferred compared to Schottky diode or transistor-based elements. Based on the comparison, as shown in Table 2, on different nonlinear elements, the potential of those elements for passive harmonic RFID fabrication is summarized in Table 3. Although the Schottky diode-based harmonic generators in Table 3 do not have good conversion loss or switching control, they still qualify over the transistor-based harmonic generators due to almost no DC power consumption, which is an essential criterion to qualify for passive RFID tags.

## 4. Enhanced Harmonic Generation: NLTL

**Figure 6:**
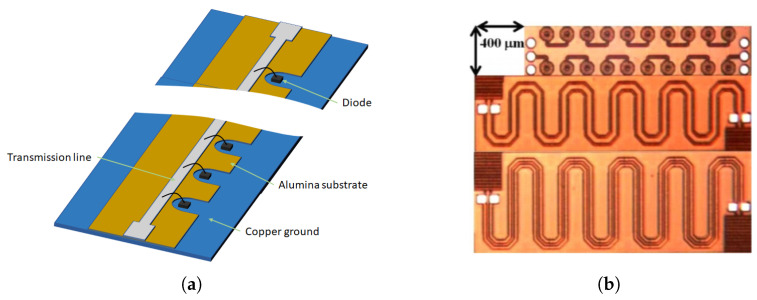
(**a**) Example of nonlinear transmission line (NLTL): (**a**) Discrete diode with co-planar waveguide (CPW) line [54]. (**b**) Discrete inductors and diodes shown at the top [38] [Reproduced with permission from F. Yu, A novel passive RFID transponder using harmonic generation of nonlinear transmission lines; published by IEEE, 2010].

In the previous section, the building block component for harmonic generation is discussed. This section will show how the harmonic generation efficiency can be further enhanced by using multiple of those building block elements. The generated harmonic signals from multiple sections are added in-phase to generate very low conversion loss harmonic output. One such structure is called the nonlinear transmission line (NLTL). The NLTL can be a continuous co-planar waveguide (CPW) line on a semiconductor substrate with discrete metal-semiconductor junction diodes [52,54], or distributed configuration using a metal-semiconductor junction on GaAs substrate [55], or discrete inductor and varactor diode structure fabricated monolithically [38], or discrete inductor and varactor diode structure fabricated on a PCB using discrete packaged components [56]. As the maximum bandwidth is dictated by the NLTL cut-off frequency, a monolithic fabrication would provide the most operational bandwidth as high as 20–30 GHz [54]. Examples of differently fabricated NLTLs are shown in Figure 6, for (a) discrete diodes on the CPW line, and (b) discrete diodes and inductors.

### Design Principle

**Figure 7:**
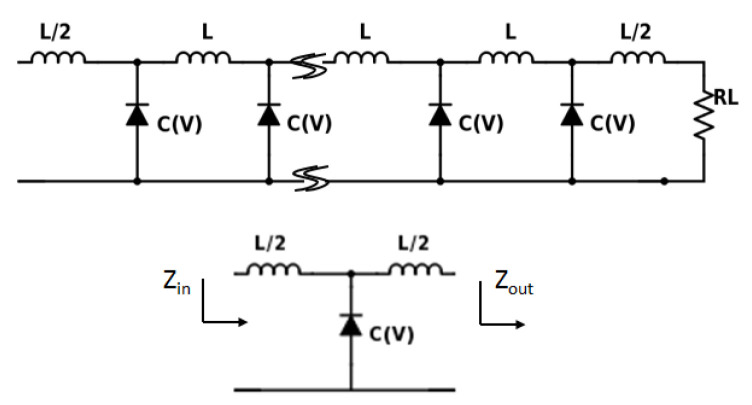
Equivalent circuit of multiple section NLTL and a single section NLTL with input and output impedance.

The design principle of NLTL is described in detail in [27,54,56] with the periodic structure of multiple sections of NLTL. There are different methods of designing NLTL circuits, among which the prevalent one is to approximate a single section as an equivalent LC network similar to a transmission line but with nonlinear capacitance due to diode instead of constant capacitance. A single and multiple section NLTL is shown in Figure 7. In standard design methodology, three parameters are very important: (a) nonlinearity of individual diode, (b) the line impedance of the NLTL, and (c) the number of NLTL stages. The nonlinearity of individual diode elements was demonstrated in the previous section. The line impedance is maintained by the *L* and *C* values of the NLTL, and the maximum cut-off frequency of the NLTL is ensured by maintaining the frequency independence of those components at as high a frequency as possible. The line impedance $Z_{NLTL}$ dependence on *L* and *C* parameters is expressed as in (3) for a lossless line [27].

(3) $$\begin{array}{c} \frac{L}{C(V)}-\frac{\omega^{2}L^{2}}{4}=Z_{NLTL}^{2} \end{array}$$

The telegrapher’s equations for NLTL were solved in [49] to express the output at the harmonic frequency in terms of nonlinearity factor and input at the fundamental frequency. As mentioned earlier, the number of NLTL stages is important as it decides the maximum harmonic output power available. Primarily two major events happen in NLTL: (a) the output harmonic power is added in-phase as the input propagates through the NLTL, and (b) attenuation of both harmonic and input power as the physical NLTL is lossy in nature. The optimum number of stages or the NLTL length would be based on these two contrasting factors until the harmonic generation is more than attenuation. As a good practice, the NLTL attenuation should be kept as low as possible for maximum harmonic output power. Another way to understand the NLTL phenomenon of in-phase output power addition is from the optical regime, where nonlinear parametric mixing is used extensively [57,58]. In parametric mixing, a high power pump signal gets divided into a low power idler signal and the different term as desired mixing output. When the idler is exactly at twice the frequency of pump frequency, a harmonic generator is obtained.

Due to efficient harmonic generation and very low DC power consumption, nonlinear varactor diodes are arranged in NLTL configuration and proposed as an effective harmonic generator for harmonic RFIDs. Apart from harmonic generation efficiency, the wireless propagation loss is equally important to realize an effective harmonic RFID. According to the Friis transmission equation, the propagation loss increases with frequency and hence a low frequency operation is desired for long range. Simultaneously, the tag size increases with lower operating frequency due to the larger size of an efficient antenna. Hence, a tradeoff should be maintained between range and tag dimension while designing a harmonic RFID tag.

## 5. Harmonic RFID

**Figure 8:**
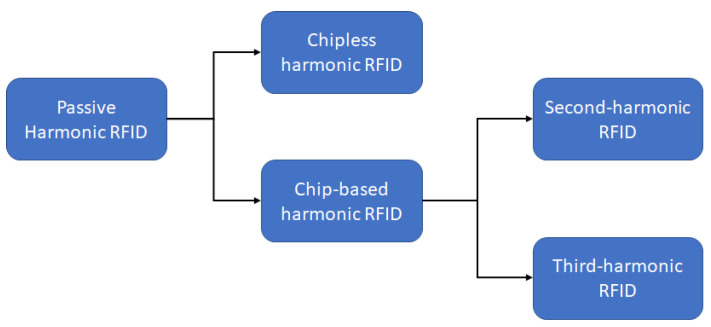
Recent trend in passive harmonic RFID systems.

The methodology to generate low conversion loss and an efficient harmonic are discussed in detail in the previous sections. In this section, the different methods to realize a complete harmonic RFID tag using those harmonic generators will be discussed. Similar to the conventional RFID system, there are primarily two types of harmonic RFID: (a) chipless harmonic RFID, where the ID bits are encoded in the frequency domain, and (b) chip-based harmonic RFID, where the ID bits are encoded in the time domain. The advantage of chipless harmonic RFID is the simplicity of the circuit. However, the bandwidth requirement is significantly large for chipless harmonic RFIDs with a higher number of ID bits. On the other hand, the chip-based harmonic RFID with ID bits in the time domain has a significantly complex circuit, but the bandwidth usage is extremely low. The trend in passive harmonic RFID systems is summarized in Figure 8.

### 5.1. Chipless Harmonic RFID

One-bit to multi-bit chipless harmonic RFID tags have been proposed earlier [59,60]. The chipless harmonic tags consist of two antennas resonating at fundamental and harmonic frequencies and a Schottky diode-based harmonic generator. In [59], the one-bit tag used a Schottky diode, HSMS-2850, for harmonic generation. The tag used two antennas at the fundamental and harmonic frequencies for signal reception and reply from the harmonic RFID reader. A read range of approximately 1 m was achieved using this setup. In [60], a multi-bit harmonic RFID tag was proposed using BAT 15-03 Schottky diode and resonators in different configurations. One configuration was to use split-ring resonators as band stop filters at the desired frequency spectrum to encode the ID. A wideband antenna was required to cover the complete bandwidth of operation. Another configuration was to use multiple dipoles resonating at the desired frequency band pairs, fundamental and harmonic. One such dipole pair represents one-bit in this configuration. As the number of bits is increased, the required number of antennas increases significantly, making 2n antennas a requirement for *n* bits. Other chipless harmonic RFID work exploited the idea of information encoding within the phase information [61]. In this work, the proposed tag first generates the harmonic of the received signal then splits it into two orthogonal channels. The orthogonal channels were realized using two orthogonally polarized antennas.

**Figure 9:**
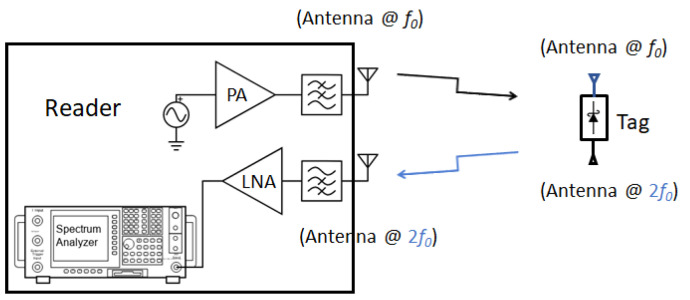
Schematic diagram of a chipless harmonic RFID tag and reader system with two antennas.

Although the chipless tags have a very simple architecture, most of them have the fundamental limitation of the number of ID bits encoding and many proof-of-concept works demonstrate only single bit encoding [29,62,63]. Likewise, the harmonic RFID reader architecture is also simple in nature. The harmonic reader primarily contains a transmitter at fundamental frequency and a power level detector (like spectrum analyzer) at the desired harmonic frequency. A schematic of a chipless harmonic RFID system with a tag and reader is shown in Figure 9.

### 5.2. Chip-Based Harmonic RFID

Apart from chipless harmonic RFID, other works on harmonic RFID are based on chip integrated RFID. In addition, under chip integrated RFID, there are primarily two lines of work: (a) second harmonic RFID and (b) third harmonic exploitation of conventional RFID. First, the second harmonic RFID system is described, followed by harmonic RFID using conventional RFIDs.

#### 5.2.1. Second Harmonic RFID

**Figure 10:**
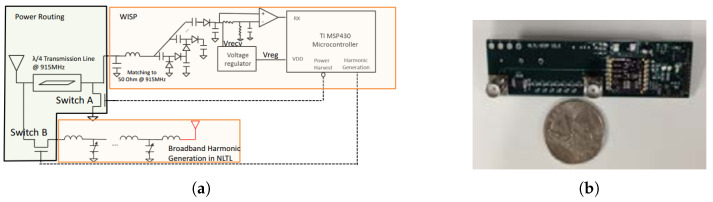
(**a**) Schematic block diagram of the harmonic RFID, and (**b**) implementation of the harmonic RFID circuit as in [25]. [Reproduced with permission from Y. Ma, A passive broadband harmonic RFID platform; published by IEEE, 2016].

This type of harmonic RFID is a balanced integration of functional units of conventional chip-based RFID and harmonic generators. Conventional RFID uses fundamental functional blocks as: (a) energy harvester, (b) digital circuit for ID generation, and (c) antenna. In a harmonic RFID system, an additional building block of a harmonic generator is added. Furthermore, as harmonic RFID uses two frequency bands for communication, multi-band or multiple antennas are required for a harmonic RFID tag and reader. Different harmonic RFID configurations were proposed in [24,25,64]. In [25], a fully functional first harmonic RFID tag was proposed. The proposed tag used an NLTL-based harmonic generator and operated within the 700 to 1200 MHz band for the fundamental frequency. A schematic block diagram of the tag circuit and implementation are shown in Figure 10. The proposed harmonic RFID tag requires two broadband antennas for NLTL impedance matching at fundamental and harmonic frequencies.

**Figure 11:**
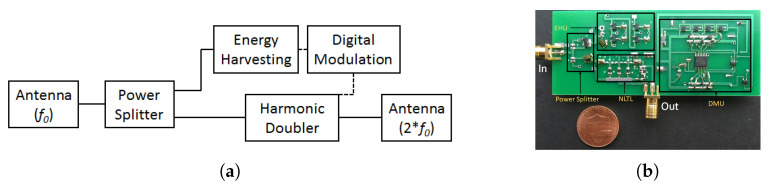
(**a**) Schematic block diagram of the harmonic RFID, and (**b**) implementation of the harmonic RFID circuit as in [24]. [Reproduced with permission from S. Mondal, A passive harmonic RFID tag and interrogator development; published by IEEE, 2019].

In [24], an 8-bit harmonic RFID tag was proposed for a 434 and 868 MHz frequency duplex as the fundamental and harmonic frequency, respectively. Similar to [25], Ref. [24] also required two antennas. However, in [24] broadband antenna was no longer required and hence the harmonic RFID tag could be miniaturized with small feature size antennas both at input and output. The schematic block diagram and the circuit implementation of harmonic RFID are shown in Figure 11.

**Figure 12:**
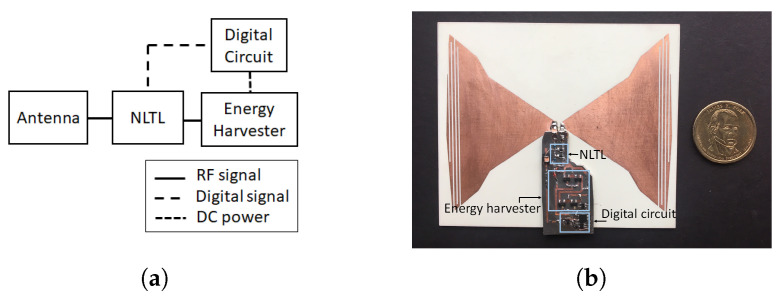
(**a**) Schematic block diagram of the harmonic RFID, and (**b**) implementation of the harmonic RFID circuit as in [64]. [Reproduced with permission from S. Mondal, A continuous-mode single-antenna harmonic RFID tag; published by IEEE, 2020].

The most recent harmonic RFID tag reported in [64] also operates on 434 MHz and 868 MHz frequency duplex as fundamental and harmonic frequency. The reported harmonic tag uses a dual frequency band single antenna and hence greatly reduces the tag real estate area [65]. The tag architecture also reduces the number of components compared to [24,25]. In addition to standard conventional RFID components, the only extra component required in [64] is the harmonic generator. To the best of our knowledge, the harmonic RFID tag reported in [64] will require the least tag area for the same frequency operation among the reported chip-based second harmonic RFID tags. The schematic block diagram and the circuit implementation of harmonic RFID are shown in Figure 12. In addition to miniaturized tag dimension, the harmonic RFID can harvest energy at both modulation states, unlike conventional RFID, which cannot harvest energy when the antenna is shorted.

The second harmonic RFID reader can be a heterodyne or a homodyne design. A heterodyne receiver architecture was proposed in [66], where the reference clock is required to demodulate the tag signal. In [24], a homodyne receiver architecture was proposed for tag signal demodulation. The antenna and filter system at the receiver operates as a bandpass filter (BPF) to prevent undesired signal effectively at the demodulated output for homodyne receiver.

#### 5.2.2. Harmonic Exploitation of Conventional RFID

**Figure 13:**
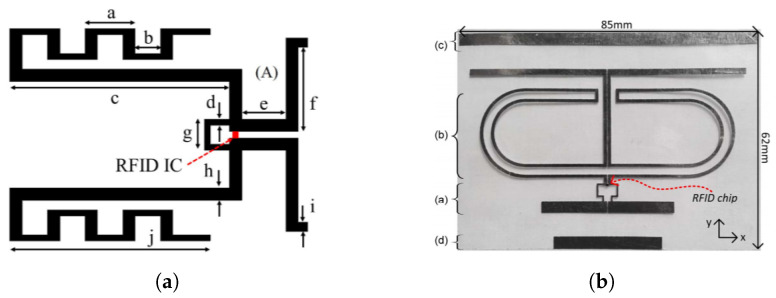
Harmonic RFID tag realization using conventional RFID IC (**a**) as in [73], [Reproduced with permission from D. Kumar, Harmonic RFID communication using a conventional ultra high frequency (UHF) system; published by IEEE, 2019.] and (**b**) as in [74]. [Reproduced with permission from G.A. Vera, Third harmonic exploitation in passive UHF RFID; published by IEEE, 2015].

In parallel to the second harmonic RFID system development, another popular approach was to exploit the third harmonic generation from the conventional RFID tag [67,68,69,70,71,72,73,74]. In this method, the antiparallel combination of detector diodes in a charge pump configuration was leveraged towards third harmonic generation from a conventional RFID. A conventional RFID tag would backscatter modulated the third harmonic along with the modulated fundamental carrier. In the literature, the generated third harmonic is exploited in two ways: (a) harvest the third harmonic and enhance the read range [71,72]; and (b) use the third harmonic for tag-to-reader link communication [70,73,74]. The implementation of the conventional tag and backscattering third harmonic is shown in Figure 13. A dual band antenna, resonating at both the fundamental and third harmonic, is required for third harmonic exploitation of the conventional RFID tag. In [73,74], impedance matching of the antenna was performed for maximum power transfer at both frequencies. The implemented conventional tag in [73] demonstrated digital data transfer at the harmonic for the first time. A comparable read range was achieved when compared to the same tag IC used at the fundamental frequency implementation. Redesign of the tag IC for harmonic generation will allow for enhanced read range.

**Figure 14:**
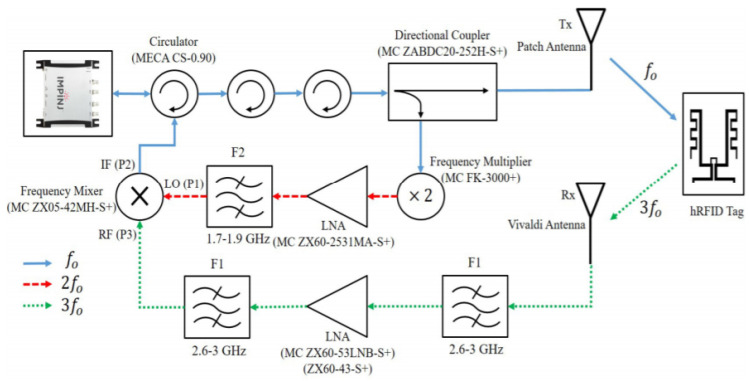
Harmonic RFID reader realization using conventional RFID reader as in [73]. [Reproduced with permission from D. Kumar, Harmonic RFID communication using conventional UHF system; published by IEEE, 2019].

The conventional reader’s RF front end needs to be modified to obtain data modulation from the tag at the third harmonic. The new reader configuration was proposed in [73], as shown in Figure 14. The primary challenge in the reader implementation was to prevent leakage of tag transmitted data modulation at the fundamental frequency. Hence, multiple circulators were used to provide enough isolation for the backscattered fundamental frequency. Additionally, the transmitted fundamental frequency path and received third harmonic frequency path were separated using two antennas. The tag modulated third harmonic signal was downconverted at the reader using the second harmonic reference signal. The conventional RFID reader could successfully decode the tag ID from the downconverted fundamental signal.

### 5.3. Antenna Design for Harmonic RFID

One of the major design challenges of harmonic RFID implementation is the dual frequency antenna design. One objective of harmonic RFID is to miniaturize the overall tag area, which requires small antennas, and simultaneously maintain a considerable gain at both the frequencies. Many different antenna designs were proposed in the literature for dual frequency harmonic RFID tag design. In [75], two nested annular slot antennas were used at a 1.2 and 2.4 GHz frequency duplex. As the 2.4 GHz antenna is nested within the 1.2 GHz antenna, the maximum tag area would be the area occupied by the 1.2 GHz antenna. In [63], two half wavelength patch antennas were used at 868 MHz and its second harmonic duplex. As both the patch antennas were kept side-by-side, the total tag area would be at least the summation of both antenna areas. In [29], parallel connected dual band slot antennas resonating at 2.5 and 5 GHz were proposed. In [65], an edge slotted bow-tie antenna was proposed. The bow-tie antenna was designed for second harmonic frequency, and the edge slot provided a secondary resonance for fundamental frequency. Other harmonic tag implementations using conventional RFID ICs required dual antennas resonating at fundamental and third harmonic frequency. In [73], a meandered dipole at fundamental frequency and a standard dipole at third harmonic frequency were implemented. The meandering at the fundamental frequency reduced the overall tag dimension.

## 6. Applications

Different applications of harmonic RFID reported in the literature can be broadly categorized into three primary categories as mentioned below. Each of the applications is described in detail subsequently.

- High resolution ranging and vital monitoring,
- Tagging and physical parameter sensing,
- Harmonic radar.

### 6.1. High Resolution Ranging and Vital Monitoring

In [35,37,76], ultra-high resolution in the range of millimeter to micrometer was reported using broadband harmonic RF ranging. In [76], a second harmonic backscattering solution was proposed with millimeter scale accuracy, even effective against body motion interference. In [37], a 1 GHz harmonic RFID system was used to demonstrate a ranging resolution of less than 50 micrometers with a sampling rate of greater than 1 kHz.

Ultra-high resolution of harmonic tags has enabled detecting very slight mechanical motion resulting from heartbeats or movement from arterial vessels. In [28], vital sign monitoring such as ECG and heart rate monitoring was demonstrated using non-contact harmonic tags. In [77], a long-term sleep monitoring system was proposed using a harmonic RFID system. The system used a harmonic near-field coherent sensing (NCS) to monitor the heart rates, breath rhythm, and body motion. Different motion classification and sensing using semi-supervised learning while sleeping can provide more insight into a patient’s recovery or sleeping quality.

### 6.2. Tagging and Physical Parameters Sensing

Buried asset tagging in utilities such as underground pipes was proposed in [29,30,63]. Tagging of pipelines helps in reducing localization error and hence minimizing the damages during excavation. The dual frequency harmonic RFID tagging is specifically advantageous for underground object tagging as clutter becomes a primary drawback for RF interrogation. Additionally, a broad range of non-contact physical parameter sensing methods using harmonic RFID systems has been proposed in the literature. The reported sensors are gas, vibration, humidity, temperature, crack, pressure, pH, etc. Those sensors’ fundamental methodology is to convert the physically realizable parameters into electrical signals and couple that either to power or phase of the RF interrogation. Finally, the harmonic tag backscatters at the harmonic of the RF interrogation and the sensor signal is interpreted from the tag reflected harmonic power and phase information.

A CNT-based gas sensor was proposed in [78], operating at 868 MHz and its second harmonic duplex. The CNT-based harmonic sensor was demonstrated for ammonia sensing in Large Area Electronics (LAE) applications. In [79], a harmonic RFID-based vibration sensing system (TagSound) was proposed that explores a tag’s harmonic backscattering to recover high-frequency (>1 kHz) and tiny mechanical vibrations (<2 mm) accurately. Commercial RFID tags were used to demonstrate the concept and the system works at 920 MHz and its third harmonic.

In [80], a harmonic humidity sensor was proposed, where the harmonic antenna is loaded with an interdigital capacitor with humidity-dependent capacitance. The antenna gets detuned as the humidity varies, and hence the received second harmonic power is changed according to the humidity. The system works at 868 MHz and its second harmonic duplex. In [81], a harmonic RF tag was proposed for temperature sensing. The received signal at the tag is first passed through a filter, which is temperature dependent, and then the converted second harmonic signal is transmitted back to the reader. The system works at 1.59 GHz and its second harmonic duplex. In [59], a harmonic RFID tag was proposed for crack sensing. The crack sensor was implemented by adding a disposable band-stop filter on cellulose substrate. The system works at 1.04 GHz and its second harmonic duplex.

In [82], a harmonic RF tag system was proposed for pressure sensing in buried plastic pipes. The sensor was implemented using a membrane based pressure sensor coupled to a phase shifter. The sensor tag converts the pressure information into phase data and changes the phase of the interrogation signal. The system works at 2 GHz and its second harmonic duplex. A similar system was also proposed in [83] for remote monitoring of a pH sensor.

### 6.3. Harmonic Radar

Another important application of a harmonic RF system is harmonic radar implementation for electronically tagged object tracking. The harmonic radar system is advantageous for habitat tracking of bees and insects [2,84,85,86,87]. As the tags are worn by the bees or insects, a very small form-factor harmonic tag is required. The simplest form of those harmonic tags was implemented using a dipole antenna with center-loop and a Schottky diode for a harmonic generation. The fundamental frequency and interrogation range reported in harmonic radars are given as 9.375 GHz and 200 m in [2], 5.96 GHz and 58 m in [84], 2.9 GHz in [85], 9.41 GHz and extendable to 200 m in [86]. A more comprehensive study of harmonic radars for tracking bees can be found in [87].

Other notable works of harmonic radar are to enable first-responders to pinpoint personal electronics during emergencies such as immediately after an avalanche or earthquake and initiate rescue [88,89]. Furthermore, the harmonic radar exploits the nonlinear characteristics of RF electronic devices, which would help law enforcement agents to locate devices of which the emissions exceed those permitted by law and allow security personnel to detect unauthorized radio electronics in restricted areas [90].

## 7. Trends and Challenges in Harmonic RFID

**Figure 15:**
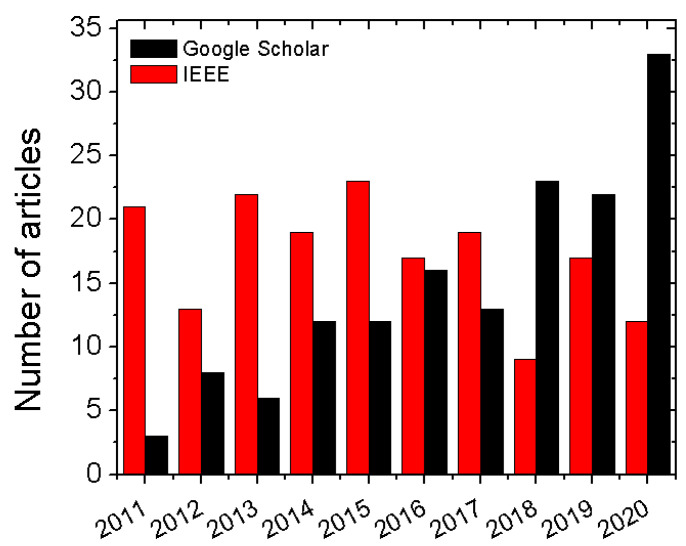
Number of relevant articles over the years as a search result.

The relevance and popularity of a research topic can be measured by the number and growth of research articles published in previous years. To quantify the popularity of harmonic RFID, ‘Harmonic RFID’ was used as the search input in Google Scholar and the IEEE database. The yearly breakdown of relevant search results for the last ten years is shown in Figure 15. In Google Scholar, the exact phrase search was performed to exclude other non-relevant results, and this process may exclude a few relevant articles that did not use the specific phrase. However, the IEEE search engine considers ‘harmonic’ and ‘RFID’ as two different search keywords. Hence, many of the results may not be relevant. Nevertheless, the Google Scholar data trend in Figure 15 shows that harmonic RFID is comparatively a new research topic, and its relevance is increasing yearly.

### 7.1. Security

Security in RFID-based tags is another growing concern with the increasing use of RFIDs in many applications [91,92,93]. Security issues can happen in operating system software, as well as at the hardware level. Counterfeiting of RFIDs is possible when the ID bits of a legitimate RFID are cloned into a replicated RFID. Identification of those counterfeited products is becoming more challenging with the global supply chain becoming geographically more complicated. Hence, a hardware level security is required to prevent the cloning of the tags.

Physically unclonable functions (PUFs) have been earlier proposed for hardware security in a silicon based process [94]. By leveraging the fabrication process tolerance, a hardware level PUF implementation is possible, which will be unique to a specific IC. For example, the same ring oscillator design across different chips will have different oscillation frequencies due to process variation and this phenomenon can be used as a fingerprint of the chips consisting of the oscillator. In short, PUF can be regarded as an IC, capable of providing different outputs for the same set of inputs when implemented on different chips. Integrating the digital level PUF technology along with RFID IC will provide a hardware level security and prevent cloning of RFID ICs. Different PUF implementation for RFID systems has been proposed in the literature [93,95,96]. However, implementing PUFs in an RFID IC will consume additional power at the RFID IC, which can impact the read range. In regards to that, harmonic RFIDs will have an in-built hardware fingerprint. Using the concept of process tolerance, the multiple order harmonics originated from a harmonic RFID tag can be used as a fingerprint of a harmonic RFID [97]. Hence, no additional hardware system is required for the harmonic RFID tag to implement a PUF. Despite many advantages of harmonic RFID over conventional RFID, there are currently two major challenges: miniaturization and available frequency bands.

### 7.2. Miniaturization

The requirement of additional components in harmonic RFID compared to conventional RFID has been greatly reduced since its first inception. However, to obtain a state-of-the-art size of harmonic RFID IC, a CMOS implementation of harmonic RFID IC is required. As reported in [64], only an additional harmonic generator is required with conventional RFID components for a working harmonic RFID. CMOS implementation of NLTL as the harmonic generator was already demonstrated in [38]. Hence, it is possible to implement the complete harmonic RFID IC monolithically using the CMOS process. Apart from IC miniaturization, antenna miniaturization is another aspect of complete harmonic tag size reduction.

Antenna miniaturization is always a challenge specifically when multiple frequency resonance is desired. In conventional RFID, a high gain antenna is used at the reader for a longer read range. On the other hand, a high gain antenna resonating at both fundamental and harmonic frequency is required for a harmonic RFID reader. One way is to use an array of multiple antenna elements to realize a high gain antenna. Array implementation at harmonic frequency receiving antennas at the reader is more desired than the fundamental frequency, as the effective wavelength at harmonic frequency is smaller.

### 7.3. Frequency Bands

License-free frequency bands are a challenge for harmonic RFID. As harmonic RFID requires two frequency duplexes (fundamental and its harmonics) for communication, and availability of the frequency duplex is required for large scale harmonic RFID deployment. Currently, there is no such frequency band available in a specific country. One approach to solve the issue is multi-band communication [98]. In this communication method, more than one frequency is transmitted from the reader, and the tag mixes them and filters out the desired frequency band. For example, if the multi-band RFID reader transmits at ISM bands 900 MHz and 2.45 GHz, the tag can reply by mixing the second harmonic of 2.45 GHz and 900 MHz to reply back at 5.8 GHz. Due to nonlinearity, an NLTL-based harmonic generator can be used for the proposed multi-band communication. However, the conversion loss will be very significant in this method.

## 8. Conclusions

In this review article, the advantage of harmonic RFID over conventional RFID has been analyzed and discussed in terms of reduced self-jamming, location accuracy, and improved SNR. Furthermore, recent developments on harmonic RFID, along with the selection of nonlinear devices suitable for small form factor passive harmonic RFID, are presented. It is shown that harmonic RFID have a potentially in-built hardware security feature unlike conventional RFID. The primary challenges of harmonic RFID identified in this review are miniaturization and commercial frequency band availability. It was also discussed that future miniaturization is possible with the current trend and CMOS implementation of harmonic RFID. Simultaneously, a large scale deployment demand of harmonic RFID is required in the future to enable desired frequency bands by regulatory authorities.

## Figures and Tables

**Table 1:** Frequency multiplication techniques.

Ref.	Device Type	Output Frequency	Multiplication Factor	Power Consumption
[40]	CMOS	3.2 or 4.8 GHz	2× or 3×	3.7 mW @ 1 V for 2×
[41]	CMOS	2.4 GHz	2×	245 uW @ 0.7 V
[43]	SiGe BiCMOS	22–30 GHz	2×	65 mW
[44]	CMOS	5.2 GHz	2×	9 mW @ 1.8 V and −4 dBm i/p power
[39]	CMOS logic gate	1.2 GHz	8× to 10×	52.5 mW @ 2.5 V
[42]	Graphene based FET	1.4 GHz	2×	NA
[23]	Schottky diode	2.4 GHz	2×	no external bias
[29]	Schottky diode	5 GHz	2×	no external bias
[45]	Schottky diode	2 GHz	2×	no external bias
[38]	CMOS varactor diode	Up to 25 GHz	2× and 3×	negligible power
[24]	Varactor diode	868 MHz	2×	20 uW @ 0.6 V and −4 dBm i/p power

**Table 2:** Comparison of different harmonic generation techniques.

Architecture	Conversion Efficiency	Power Consumption	Cut-Off Frequency	Switching Control
Transistor	Very good	High	High (using small process node)	Easy
Schottky diode	Good (at high input power)	Zero	Very high	Difficult
Varactor diode	Good	Zero or negligible	Moderate	Easy

**Table 3:** Comparison of different multipliers for potential passive harmonic RFID candidates.

Reference	Power Consumption	Conversion Efficiency	Switching Control	Potential Passive Harmonic RFID Candidate
CMOS [40]	High	Good	Good	Weak (due to high power consumption)
CMOS [41]	Medium	Good	Good	Moderate (due to medium power consumption and good efficiency and control)
BiCMOS [43]	High	Good	Good	Weak (due to high power consumption)
CMOS [44]	High	Good	Good	Weak (due to high power consumption)
CMOS logic [39]	High	Good	Good	Weak (due to high power consumption)
FET [42]	High	Good	Good	Weak (due to high power consumption)
Schottky [23]	Low	Bad	Bad	Moderate (due to complicated switching control)
Schottky [29]	Low	Bad	Bad	Moderate (due to complicated switching control)
Schottky [45]	Low	Bad	Bad	Moderate (due to complicated switching control)
CMOS varactor [38]	Low	Medium	Good	Strong (due to all advantages)
Varactor [24]	Low	Medium	Good	Strong (due to all advantages)
